# Application of Inertial Motion Unit-Based Kinematics to Assess the Effect of Boot Modifications on Ski Jump Landings—A Methodological Study

**DOI:** 10.3390/s20133805

**Published:** 2020-07-07

**Authors:** Nicolas Kurpiers, Nicola Petrone, Matej Supej, Anna Wisser, Jakob Hansen, Uwe G. Kersting

**Affiliations:** 1 Institute of Sport Science, University of Hildesheim, 31141 Hildesheim, Germany; 2 Department of Industrial Engineering, University of Padova, 35131 Padova, Italy; nicola.petrone@unipd.it; 3 Faculty of Sport, Lljubljana University, 1000 Ljubljana, Yugoslavia; matej.supej@fsp.uni-lj.si; 4 Sport Sciences, Aalborg University, 9000 Aalborg, Denmark; anna.wisser@stud.pmu.ac.at (A.W.); Jaohan@rm.dk (J.H.); u.kersting@dshs-koeln.de (U.G.K.); 5 Institute of Biomechanics and Orthopaedics, German Sport University, 50933 Cologne, Germany

**Keywords:** skiing, kinematics, motion analysis, footwear, measurement systems

## Abstract

Biomechanical studies of winter sports are challenging due to environmental conditions which cannot be mimicked in a laboratory. In this study, a methodological approach was developed merging 2D video recordings with sensor-based motion capture to investigate ski jump landings. A reference measurement was carried out in a laboratory, and subsequently, the method was exemplified in a field study by assessing the effect of a ski boot modification on landing kinematics. Landings of four expert skiers were filmed under field conditions in the jump plane, and full body kinematics were measured with an inertial motion unit (IMU) -based motion capture suit. This exemplary study revealed that the combination of video and IMU data is viable. However, only one skier was able to make use of the added boot flexibility, likely due to an extended training time with the modified boot. In this case, maximum knee flexion changed by 36° and maximum ankle flexion by 13°, whereas the other three skiers changed only marginally. The results confirm that 2D video merged with IMU data are suitable for jump analyses in winter sports, and that the modified boot will allow for alterations in landing technique provided that enough time for training is given.

## 1. Introduction

Freestyle skiing has enjoyed increasing popularity since the inclusion of ‘Slope Style’ at the Winter Olympic Games in 2014. Consequently, ski resorts nowadays provide parks with obstacles, rails and table top kickers that are increasingly frequented by young recreational skiers. Unfortunately, the domain of freestyle skiing is also associated with an alarming number of injuries, largely affecting the knee joint [1]. Flørenes and colleagues [2] found that about 50% of all competitive World Cup freestyle skiers suffered injuries in the seasons from 2006 until 2009, a third of which were severe and involved the knee joint. Three major injury mechanisms are known for anterior cruciate ligament (ACL) ruptures, one of which is the so-called boot-induced anterior drawer (BIAD). This injury mechanism mostly occurs in landing situations with a leaning back tendency as is oftentimes prevalent in mogul skiing or slope style. The boot shaft is pushing forward on the tibia while the body weight is falling backward, thus applying harmful force to the ACL. The limited range of motion of conventional ski boots is believed to favor this highly critical position when bending the legs in order to absorb a bump or to decelerate the upper body in a landing situation [3,4,5].

Field tests in skiing face the challenge of collecting high-quality data under difficult environmental conditions. Previously, studies have shown that video-based data collection is comparable to laboratory standards [6,7,8,9], while the logistics of installing multiple cameras in a ski field pose some limitations on the practicalities of such studies. A possible circumvention of this difficulty is the use of inertial motion unit (IMU) -based kinematic measurements in conjunction with differential global navigation satellite systems (GNSS), which provide a spatial resolution of a few millimeters [10]. Similar approaches have been utilized previously for slightly different applications [11,12,13,14,15,16,17,18]. We propose that a combination of 2D video recordings with IMU-based kinematics will provide a feasible solution for the assessment of full body motion, for example, in the evaluation of ski jump biomechanics in terrain parks.

For the current study, the same ski boot modification was utilized as in a previous investigation [7] which may have a potential effect on landing kinematics after a straight jump from a kicker. Hypothetically, the modified boot should allow for a natural squatting movement, in contrast to conventional ski boots, that usually restrict ankle motion which can force the skier into a backward position when landing, potentially leading to the BIAD injury mechanism. Previously, the influence of the tested boot on knee joint loading was positively evaluated in a mogul course using a full body computer model (Anybody Modelling Software, Aalborg, Denmark). The calculated anterior–posterior tibia forces were reduced by almost two thirds, from 4855 N to 1793 N [7].

The goal of the current study was twofold. First, it aimed at evaluating a measurement method where 2D video data and sensor-based full body motion capture are merged in a laboratory experiment. Second, the effect of ski boot shaft flexibility on jump landing kinematics with a focus on ankle and knee angles was assessed. It was hypothesized that maximum knee and ankle joint flexion would increase with the flexible boot.

## 2. Methods

In the laboratory study, two participants (1/1 female/male, 24/25 years) where equipped with an IMU-based motion capture suit with 17 sensors (240 Hz, Xsens MVN, Enschede, The Netherlands) and a full body marker set (53 retroreflective markers) recorded by eight cameras (240 Hz, Oqus 3 series, Qualisys, Gothenburg, Sweden). The experiment included a selection of six simulated snowboard landings in total in a laboratory setting, e.g., jumping off a box onto a mattress, jumping with the snowboard from a moving roller board or from a swing with a climbing rope, releasing and landing on the mattress. For this part of the study, we chose to use a snowboard, as performing the movements indoors with skis was deemed to be too cumbersome and risky. As the IMU system outputs the body movement in a local coordinate system, i.e., ‘hip fixed’, one marker from the Qualisys Track Manager (QTM) data was used to simulate a single point which could be gathered using a complementary system like a differential GPS [10] or, as in the second part of this paper, a 2D video recording. The single point data were merged with the IMU data to compile a new ‘.c3d’ file in MatLab (2017, The MathWorks, USA) using the Biomechanical Tool Kit (BTK, downloaded from http://biomechanical-toolkit.github.io on 03/03/2016) and compared to the motion data from the Qualisys recording. Both were applied to a full-body model in Visual3D (C-motion, Germantown, MD, USA) to calculate joint angles (ankle, knee, hip, pelvis-trunk) and center of mass (CoM) position.

In the outdoor experiment, four expert skiers (ranging from former semi-professional Europe Cup competitors in freestyle skiing to otherwise freestyle competition-experienced on a regular basis) were equipped with the motion capture suit with 17 sensors measuring the knee and ankle angles continuously; then, the maximum value within two seconds after landing (Joint angle definitions: the angles of the knee and the ankle are smaller in flexion and larger in extension) was recorded. Data were transferred via a wireless network connection to the recording computer and stored on a hard disk after each trial. All subjects were informed about the testing procedure (age: 30.5 ± 7.0 years, height: 181.5 ± 5.1 cm, mass: 80.8 ± 2.2 kg; mean ± standard deviation) and gave consent according to the ethics guidelines of Padova University. The investigation was carried out following the rules of the Declaration of Helsinki.

A commercially available ski boot (Head Raptor Super Shape, Head Inc., Kennelbach, AT with a stiff shaft (ST) and a modified boot of the same model with a very flexible shaft (FL) were compared [7]. The ski boot’s shaft was removed and the residual material in the anterior and posterior direction was cut at the instep before the shaft was refitted. In order to gain a noticeably greater ankle flexion, only two screws per boot were used to fix the shaft at the medial and lateral connection point. To ensure the durability of the plastic around the pivot points and to prevent breakages, small aluminum plates were manufactured to reinforce the joint on the inside and outside of the shaft [19].

The jump geometry, landing area and various reference points on short sticks in the snow were surveyed by a RTK GNSS system (Leica, CH) with a reference station close to the jump. The landing area had an inclination of 18° and was situated at an altitude of 2100 m. The testing course consisted of an in-run of about 40 m and a landing area of about 20 m × 5 m, whereby the jump distance was about 17 m. Four reference points, mounted to vertical bars in the center of the take-off on the kicker and the area of landing, were used as calibration points for a two-dimensional video capture of the whole flight phase (50 Hz, EOS Rebel Ti5, Canon, Huntington, NY, USA). A reference point on the skier’s helmet was tracked using Skill Spector software (video4coach, Odense, Denmark).

The participants filled out a general questionnaire, and on the day of testing, they all warmed up on both the normal slope and a variety of jumps including the test kicker using the flexible boot. The familiarization runs were variable due to poor weather conditions. After familiarization, a total of six trials were recorded per participant after a break of approximately 15–20 min during the ascent by chair-lift. The testing performance consisted of straight jumps. The first three jumps were completed using the FL boot, and after a short readjustment period, three trials were recorded in the ST condition. All participants first needed to adapt their movement pattern to the greater front flexibility of the modified boots; this was important, given that this modified pattern could not be performed with the conventional boots due to the restrictions of the stiff boot shaft.

The kinematic IMU data of the flight path were transformed by aligning the flight plane with a three-dimensional world coordinate system. This was done under the assumption that the skier was moving in a single plane during flight. The videos and IMU data were synchronized using the landing impact from the foot accelerometers and the frame of foot contact with the slope. The video tracks of the head were merged with C3D files and a custom full-body model (C-motion, Visual 3D, Germantown, MD, USA) was used for kinematic analysis.

Due to the small sample size, only descriptive statistics were used to present the results. Data of both systems were compared using RMS differences and curve correlations for the time series of the kinematic parameters.

## 3. Results

**Figure 1:**
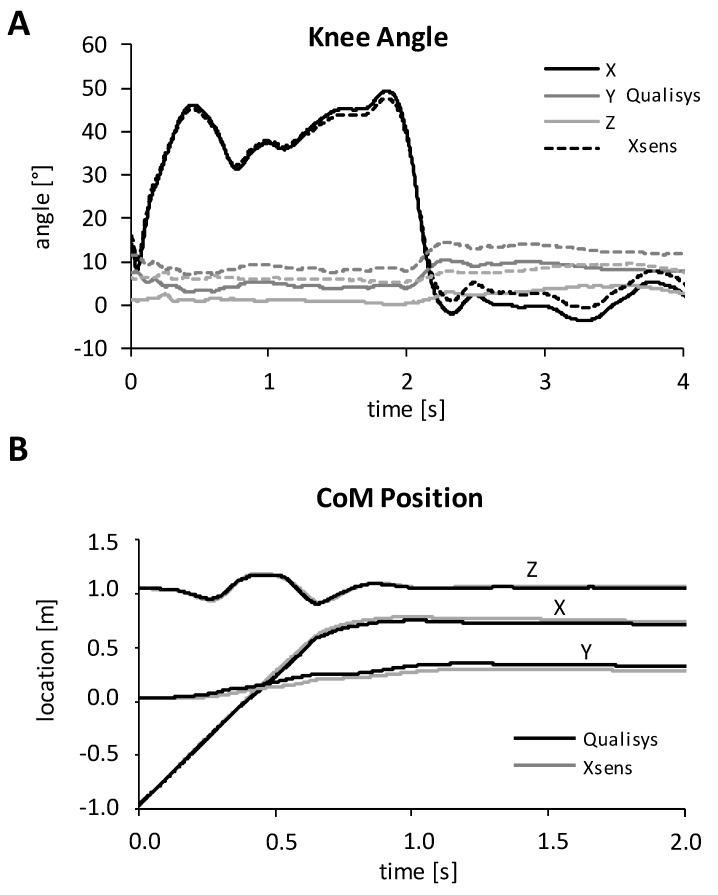
Example data of kinematic parameters from the same landing trial (**A**,**B**) as calculated using both methods. (**A**): knee angle (in the joint coordinate system of the right knee) from marker data (Qualisys), and merged IMU data (Xsens), X = flexion-extension, Y = internal-external rotation, Z = abduction-adduction. (**B**): Comparison of the Center of Mass trajectory from both methods (from a different trial than in 2A; coordinates in the laboratory coordinate system).

Laboratory study: From three simulated snowboard landing trials for each participant, the average and minimum curve correlations, as well as the RMS differences of angle and CoM position, are given in Table 1. Correlations were high for the X axis (flexion-extension), intermediate, and in some cases, low for the Y axis (internal-external rotation) and intermediate for the ad-abduction at the knee and hip or inversion-eversion at the ankle joint, respectively. Correlations for the CoM position were very high, while RMS differences were only between 0.9 to 3.1 cm between measurement systems and models (Table 1, Figure 1).

**Figure 2:**
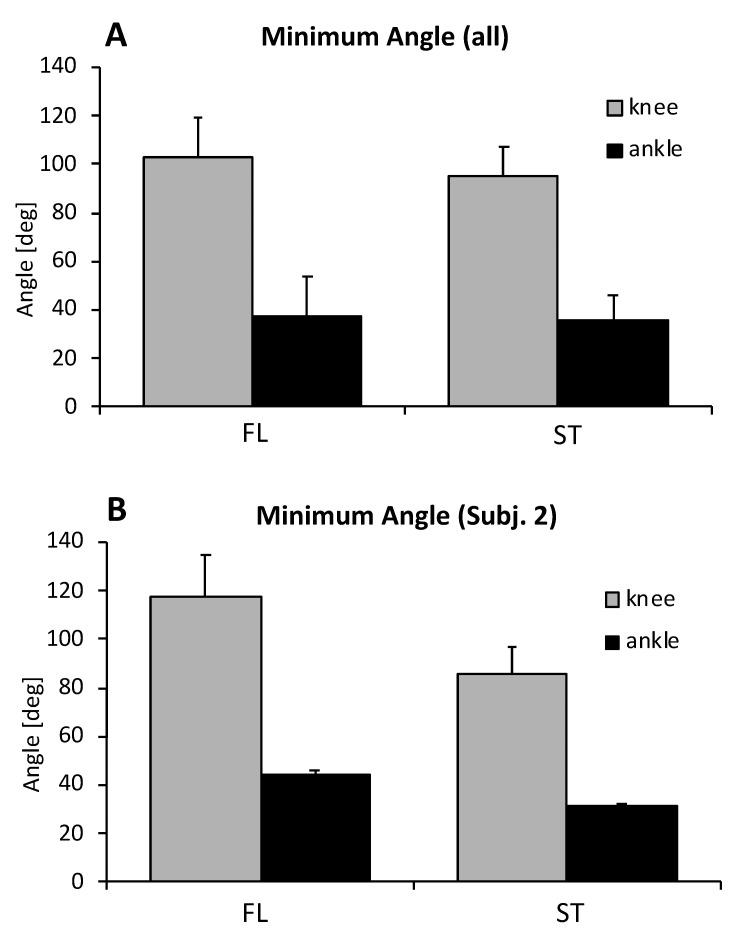
Minimum knee and ankle angle following initial contact. (**A**): Average of four subjects, (**B**): Average of three trials for Subject 2 (ankle dorsiflexion designated as positive values).

Outdoor experiment: The kinematic parameters showed only small variations for the knee flexion angle after landing, which slightly increased from the ST to the FL condition, i.e., from 95° to 103° (Figure 2A). The change in the maximum ankle flexion angle was even smaller on average, with the ST condition at 37° and the FL condition at 35° (Figure 2B).

Screening the individual data revealed that participant No. 2 showed a large change in knee and ankle angles between conditions. In this case, the knee angle was 118° in the FL boot and 85° in the ST boot, while the ankle angle was 44° in the FL boot and 31° in the ST boot condition. This participant had the longest familiarization time with the FL boots, i.e., two days, compared to 1 to 2 h for the other participants.

## 4. Discussion

In this study, a measurement method was developed and evaluated by comparing marker-based kinematics and merged IMU-based data (with one anatomical landmark; in this case, the center of the head was chosen). Kinematic data derived from these two approaches agreed well in a laboratory comparison with simulated snowboard landings. The field application of a modified ski boot condition showed only small average effects, but promising potential, as one subject was able to substantially alter landing kinematics.

The merging of the 2D video data and IMU based kinematics provided a three-dimensional data set for each jump, providing a full description of body kinematics during take-off, flight and landing; the latter is the focus of this study.

The results of the system comparison are in accordance with previous studies validating IMU-based kinematics (for the Xsens system) using marker-based recordings under laboratory conditions. Values for differences between flexion-extension angles were estimated to be below 5° [20]. Correlations ranged from 0.96 for flexion extension but with substantially lower relationships for the other two joint axes (low: 0.3–0.5; two observations, moderate: 0.5–0.7; two observations, high-very high: all others, Table 1) [21]. A possible reason for these deviations is the differences in the definition of the joint coordinate systems using both approaches. In the present study, the markers associated with the same anatomical landmarks were used to define the respective body model in C-motion. However, for the marker-based model, optical markers were used while the IMU-based analysis relied on virtual markers created by the MVN software, which were derived from the fusion algorithm employed in an unpublished algorithm. In summary, the correspondence of the kinematic results between the two systems was in the same range as previously reported [20,21]. The larger deviations observed for the CoM position (Table 1) were very close between the two approaches, while the deviations in Y-direction were the largest. In the laboratory setup, the Y-Z plane was aligned with the trunk flexion-extension plane during the snowboarding jumps. The magnitude of the Y-Z plane may be attributed to the different trunk models used. Overall, these data provide evidence that the suggested approach to analyzing winter sport data by merging Xsens recordings with precise positioning data of one single point on a skier [10] is a viable approach.

In this feasibility study, only jumps with movements in the sagittal plane were investigated. This approach can be extended to jumps including rotational elements, as the IMU-based data provide information on segment orientations and the body’s relative position of the CoM. Given the fact that the relative CoM position can be determined from a full-body anthropometric model, it could be used as a reference for the transformation between real-life and 2D camera coordinate systems. 

In this study, the reference and calibration points were surveyed using a differential GNSS system which is able to record precise measurements of the surface of the kicker and landing area. This can be realized by simpler methods when such a system is not available. This means that 3D assessments of ski and snowboard jumps become possible using an IMU suit and a single video camera in conjunction. Most importantly, the skiers would not have to carry a sensitive and heavy GNSS sensor in a backpack, avoiding damage in cases of unsuccessful jumps or hindering the execution of difficult aerial maneuvers. Furthermore, it would be unlikely to maintain good signal quality, as the GNSS antenna would be turned away from the direction of the satellite signals during aerial maneuvers.

**Figure 3:**
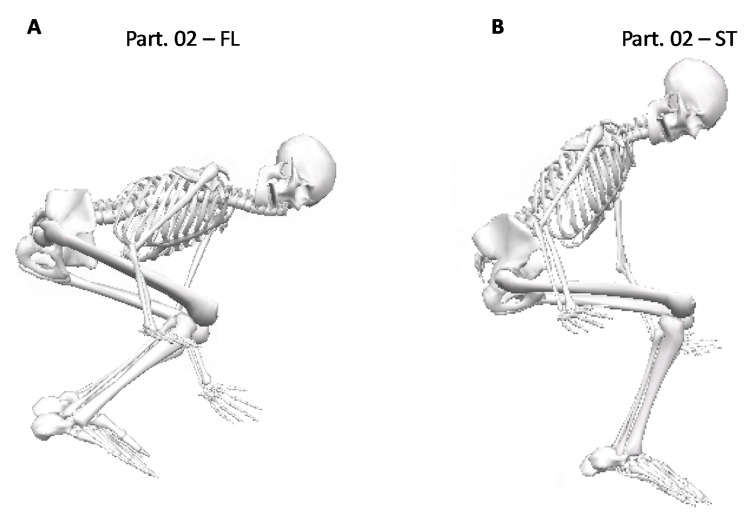
Landing positions of participant No. 2: (**A**) flex condition with modified boot, (**B**) stiff condition with conventional boot.

The mean values of the measured joint angles only changed marginally between the modified ski boot and the conventional type. An intra-individual comparison, however, showed a clear difference for participant No. 2, who was the only one that undertook two full days of familiarization training with the modified testing boot. He had approximately 50 practice runs prior to testing with the instruction to focus specifically on using the front flex in the landing situation. Without these instructions or training, the other skiers tended to maintain their usual motion patterns without using the additional flexibility, as shown in the data analysis. According to Baraduc and colleagues [22], learning skills is complex, and requires a period of consolidations after practice. The motor memory is susceptible to being disrupted by the performance of another motor-learning task; however, it remains unclear how long it takes to consolidate a new motion pattern, as this might be different from one individual to another. In any case, the full two days of training were sufficient to adopt a slightly different motion pattern and to use the greater front flex. Thus, Subject 2 changed his kinematic strategy, showing a safer landing position with the modified boot, considering that so-called ‘backseat landing’ can often lead to out-of-control situations, and thus, to injuries, e.g., through the BIAD injury mechanism [23,24,25,26] (Figure 3).

A previous study investigated a similar modified ski boot in a mogul skiing setup [7], and found an advantageous body posture with respect to the CoM relative to the ski boots and the knee joint loading at the instant of main impact against moguls; this was in agreement with the predictions of Schaff and Olbert [5]. This same study also recommended a mandatory training time of two days with the new boot, which reportedly led to kinematic and kinetic changes during mogul skiing. 

The main limitation of the current study was a lack of time for familiarization with the modified boot; as in the previous study, only one participant was able to undertake sufficient training. However, some studies have been based on single-participant-experiments, which may provide directions for future studies with greater sample sizes [27,28,29]. The one prepared participant showed a similar motion pattern as the participants of a prior investigation regarding knee and ankle joint flexion. The biggest difference between the laboratory-based study and the field test was the use of a snowboard versus skis in the landing test; these two methods are generally different in terms of landing direction, initial ground contact and impact forces. Nonetheless, this decision was made for safety reasons; ultimately, the aim of this project was not to compare indoor and outdoor landings, but to investigate the applicability of the system. Furthermore, the sample size was too small to draw any general conclusions. However, the methodological approach using the RTK GNSS system in combination with a 2D video capture system and the Skill Spector analysis software turned out to be successful and readily applicable. It has advantages compared to the use of a GNNS sensor carried by the athlete, and would make the data collection simpler and potentially safer. The application of a 2D path in a 3D world coordinate system has not been presented in the literature to date. The described approach offers an applicable and cost-efficient solution, as simple video software, such as that used in this study, is freely available.

The current study was a follow up based on the aforementioned mogul skiing investigation of the same research group; however, this application to jump landing kinematics was also a feasibility study on the proposed methodical approach. Due to the small sample size and lack of training time for some participants, the current results did not provide evidence of the proposed beneficial effect of the investigated boot design in landing situations. However, they may give pointers for optimized approaches in the future with more training time for all participants. The proposed methodology may help in creating a more substantial database of winter sport jump landings and the effects of equipment and jump design alterations.

## Figures and Tables

**Table 1:** Average and minimum correlation coefficients, range of p-values for joint angle point-by-point curve correlations (mean of three different reference movements of two participants and mean RMS differences between model calculations based on the two measurement systems (Qualisys–merged Xsens).

**Parameter**		**X**	**p_range_**	**Y**	**p_range_**	**Z**	**p_range_**
ankle angle	mean	0.923	0.000–0.04	0.378	0.08–0.24	0.818	0.007–0.06
	min	0.886		0.323		0.566	
knee angle	mean	0.988	0.00–0.001	0.566	0.002–0.14	0.841	0.007–0.013
	min	0.987		0.438		0.74	
hip angle	mean	0.991	0.000–0.008	0.665	0.01–0.08	0.967	0.01–0.04
	min	0.989		0.653		0.832	
CoM position	mean	1	0.000–0.001	0.997	0.000–0.005	0.987	0.000–0.000
	min	0.999		0.996		0.987	
**RMS**		**X**		**Y**		**Z**	
ankle angle [°]		1.2		3.6		2.5	
knee angle [°]		4.1		3.4		4.6	
hip angle [°]		3.5		3		8.9	
CoM position [cm]		1.2		3.1		0.9	
